# A Novel Capsule Network with Attention Routing to Identify Prokaryote Phosphorylation Sites

**DOI:** 10.3390/biom12121854

**Published:** 2022-12-12

**Authors:** Shixian Wang, Lina Zhang, Runtao Yang, Yujiao Zhao

**Affiliations:** 1 School of Mechanical, Electrical and Information Engineering, Shandong University at Weihai, Weihai 264209, China; 2 School of Control Science and Engineering, Shandong University, Jinan 250061, China

**Keywords:** phosphorylation, capsule network, prokaryotes, self-attention

## Abstract

By denaturing proteins and promoting the formation of multiprotein complexes, protein phosphorylation has important effects on the activity of protein functional molecules and cell signaling. The regulation of protein phosphorylation allows microbes to respond rapidly and reversibly to specific environmental stimuli or niches, which is closely related to the molecular mechanisms of bacterial drug resistance. Accurate prediction of phosphorylation sites (p-site) of prokaryotes can contribute to addressing bacterial resistance and providing new perspectives for developing novel antibacterial drugs. Most existing studies focus on human phosphorylation sites, while tools targeting phosphorylation site identification of prokaryotic proteins are still relatively scarce. This study designs a capsule network-based prediction technique for p-site in prokaryotes. To address the poor scalability and unreliability of dynamic routing processes in the output space of capsule networks, a more reliable way is introduced to learn the consistency between capsules. We incorporate a self-attention mechanism into the routing algorithm to capture the global information of the capsule, reducing the computational effort while enriching the representation capability of the capsule. Aiming at the weak robustness of the model, EcapsP improves the prediction accuracy and stability by introducing shortcuts and unconditional reconfiguration. In addition, the study compares and analyzes the prediction performance based on word vectors, physicochemical properties, and mixing characteristics in predicting serine (Ser/S), threonine (Thr/T), and tyrosine (Tyr/Y) p-site. The comprehensive experimental results show that the accuracy of the developed technique is close to 70% for the identification of the three phosphorylation sites in prokaryotes. Importantly, in side-by-side comparisons with other state-of-the-art predictors, our method improves the Matthews correlation coefficient (MCC) by approximately 7%. The results demonstrate the superiority of EcapsP in terms of high performance and reliability.

## 1. Introduction

Protein phosphorylation is a dynamic process that kinases transfer phosphate groups from adenosine-triphosphate(ATP) or Guanosine-triphosphate(GTP) to specific amino acid residues of proteins [1]. It occurs mostly at serine, threonine, and tyrosine, which normally initiates protein activities by altering protein structure [2]. As an essential protein posttranslational modification(PTM), it plays a vital role in many cell behaviors, such as DNA damage repair, transcription regulation, signal transduction, apoptosis regulation, etc.  [3]. Abnormal phosphorylation is the cause or consequence of many diseases. For example, hyperphosphorylation of the microtubule-associated protein leading to its misfolding and aggregation is a common hallmark of neurodegenerative diseases [4]. Identification of human protein phosphorylation substrates is a prerequisite for studying the effects of phosphorylation on protein molecular structure and functional activity changes [5]. It further contributes to the understanding of the pathogenesis of cancer, diabetes, etc. [6]. The regulation of phosphorylation is frequently disrupted in pathological states, and protein kinases have emerged as important targets for drug development [7]. Biochemists generally consider that protein phosphorylation is an important mode of regulation in eukaryotic cells. In contrast, the function of the protein kinase/phosphatase signaling network in prokaryotes is poorly understood [8]. But, substantial evidence demonstrates that functional phosphorylation sites in prokaryotes have a more direct effect on the phenotype than those in eukaryotes. Kinases in prokaryotes can immediately regulate biochemical pathways by altering protein function [8]. Currently, the study of bacterial resistance mechanisms and the development of new antibacterial drugs has become an urgent problem, and phosphorylation modification is a key regulatory process for bacteria in combating stressful environments [9]. Studies show that protein kinases and phosphatases from Staphylococcus aureus and Streptococcus pneumoniae contribute to pathogen viability, stress physiology, and resistance to antibiotics [10]. Regulation of protein phosphorylation appears to play a central role in key bacterial processes, which makes them major targets for therapy [8]. Therefore, accurate prediction of bacterial phosphorylation sites (p-sites) is essential to facilitate the development of new antibacterial drugs.

Despite the exceptionally rapid development of proteomics technologies, detailed and exhaustive analysis of phosphorylated proteins remains difficult through existing experimental techniques such as high-throughput mass spectrometry and low-throughput 32P label [11]. Traditional experimental methods are time-consuming and laborious, especially when verifying plenty of candidate phosphorylation sites. As a complementary technique to traditional experimental strategies, computational methods are a promising option [12].

With the accumulation of experimental data, many bioinformatics tools for phosphorylation sites have been developed. These methods can be divided into two categories, feature engineering-based methods and deep learning-based methods [13]. Most of the tools employ sophisticated feature engineering and use machine learning algorithms to judge candidate sites. For example, Xue et al., use Markov clustering algorithms to develop a kinase-specific phosphorylation site prediction method(GPS) [14]. KSP integrated protein-protein interactions(PPI) of known intraspecies and kinase-substrate relationships to predict catalytic kinases with known phosphorylation sites [15]. Salma Jamal et al., combined RF and SVM to improve the phosphorylation site prediction accuracy by introducing multiple sequences features [16]. Although the above methods have shown some progress in predicting phosphorylation sites, “feature engineering” requires human design and its limitations may lead to feature bias. Deep learning has significant advantages because it can adaptively capture high-level abstractions from raw data [17]. Based on convolutional neural networks(CNN) and bi-directional long short-term memory(Bi-LSTM), DeepPSP introduced global information of the sequence to predict the phosphorylation site. These methods using only raw sequences outperform traditional machine learning methods [18]. In contrast, PhosIDN is a deep learning system that combines sequence and PPI information to predict phosphorylation sites, further improving the accuracy of the phosphorylation site prediction [19].

Protein phosphorylation is a global regulatory mechanism [20]. However, specific phylogenetic groups have evolved different types of kinases. There is clear evidence that specific bacterial kinases have evolved independently [21,22]. In contrast to eukaryotic phosphorylation modifications, bacterial kinases are more diverse [23]. Studies have shown that eukaryotic-type Ser/Thr kinases(ESTK) in bacteria, eukaryotes, and archaea have a common evolutionary origin and may originate from a common ancestor [24]. Many serine/threonine kinases were found to lack the conserved catalytic motifs [24]. The prokaryotic-specific bacterial tyrosine kinase (BY-kinase) accounts for the majority of tyrosine phosphorylation events detected in bacteria, and it possesses a unique conserved sequence. That means calculation tools developed for human phosphorylation sites are not suitable for prokaryotes [21]. The two-component signaling system of prokaryotes is a prerequisite for some phosphorylation sites, which leads to these kinase substrates not being directly detectable in the laboratory. Computational predictors for prokaryotes can skip this limitation [24]. Despite numerous computational works in higher organisms, predictors in prokaryotic cells are still rare. To date, only a limited number of computational phosphorylation site prediction tools have been developed. Martin et al. built the first bacterial-specific protein phosphorylation predictor by artificial neural networks [25]. Zhang et al. used the amino acid position feature extraction method and support vector machine algorithm to construct predictor targets for prkC-specific phosphorylation sites  [26]. MPsite is a Random Forest (RF) based model that detects p-sites in microbial proteins by combining amino acid composition (AAC), amino acid frequency composition (AFC), and other sequence features [27].

These calculation methodologies and tools have contributed to the understanding of the mechanisms of prokaryotic phosphorylations. However, certain limitations remain. (1) The evolutionary direction of various classes of prokaryotes varies greatly. Previous methods were restricted to single organisms or single kinase substrates. (2) All prediction tools are not predictive of tyrosine phosphorylation, which is indeed widespread in the bacterial proteome. (3) The relatively small training dataset with less than 2000 samples is a bottleneck to improving the performance, and independent testing of a few dozen samples leads to large fluctuations in experimental results, which do not indicate sufficient generalization of the model. (4) Existing tools are developed using traditional machine learning techniques, and one of its limitations is that the learning algorithm is unable to send feature requirements to the feature selection algorithm.

To improve the prediction performance and address the above-mentioned limitations, a computational prediction tool EcapsP is designed by combining a self-attention mechanism and a capsule network, which automatically mines hidden high-level discriminative features without any manual feature engineering. The model outperforms the baseline hybrid neural network architecture DeepIPS and other available tools in most cases. To the best of our knowledge, deep learning-based techniques have not been applied to phosphorylation site identification in prokaryotes. To ensure the high quality of data, we merged a total of 200 experimentally validated prokaryote phosphorylation samples from dbpsp2.0. Three phosphorylation datasets are constructed for threonine, serine, and tyrosine with positive data volumes of 6629, 5029, and 3167, respectively. EcapsP trained on this benchmark dataset has the ability to predict three common phosphorylation sites (p-S/ p-T/ p-Y).

In summary, the major contributions of this study are as follows.

- A new capsule network is designed by self-attention routing, shortcut techniques, and unconditional reconfiguration decoders, to avoid weak robustness of affine transformations by the Mask mechanism and dynamic routing in CapsNet.
- EcapsP has the ability to predict the three common phosphorylation sites of prokaryotes (p-S/ p-T/ p-Y).
- EcapsP outperforms CapsNET and other predictors in most cases, especially in the case of small dataset learning.
- We investigated the multiple signature encoding modes of protein sequence fragments, including 10 types of physicochemical properties, two types of word embedding-based features, including one-hot and GloVe, and feature combinations (a combination of physical and chemical properties and word vectors).

To evaluate the performance of EcapsP, we compare it to different prokaryotic phosphorylation site datasets. The evaluation results show that EcapsP has a more accurate judgment and stronger robustness than existing methods. EcapsP is expected to facilitate the production of candidate proteins with high confidence for the discovery of novel phosphorylation events. The source code of EcapsP and the dataset is freely available at https://github.com/WangShixianChina/EcapsP.

## 2. Results

### 2.1. Amino Acid Composition Analysis of Phosphorylation Sites in Microbial Proteins

**Figure 1:**
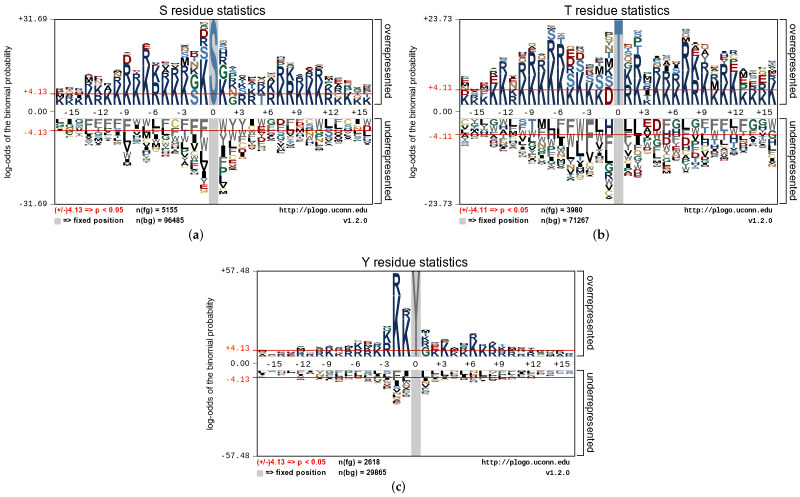
Sequence logo representation of phosphorylation. (**a**) Serine phosphorylation, (**b**) Threonine phosphorylation, (**c**) Tyrosine phosphorylation. The 16 residues upstream and downstream of the phosphorylation site are used to plot the sequence identity.

The composition of amino acids surrounding serine, threonine, and tyrosine is an essential aspect of its phosphorylation. In this study, the Plogo tool is used to display the amino acid composition at 16 positions located upstream and 16 positions located downstream of the microbial phosphorylation sites. It is a probabilistic method for visualizing protein sequence patterns, graphically labeling character heights by residue frequencies  [28]. The larger the abbreviated letters of amino acids in the upper half of Figure 1a–c, the more frequently they occur at the corresponding positions. Descriptions of over- and under-represented residues are shown above and below the X-axis, respectively. It can be observed that lysine (K) and arginine (R) residues appear frequently in the phosphorylation sites, indicating their significant roles in protein phosphorylation. Furthermore, Figure 1 clearly shows that serine phosphorylation, threonine phosphorylation, and tyrosine phosphorylation have very different patterns. This reveals that there is a significant difference in evolutionary conservation among serine phosphorylation, threonine phosphorylation, and tyrosine phosphorylation. Therefore, it is reasonable to develop separate predictors for serine phosphorylation, threonine phosphorylation, and tyrosine phosphorylation.

### 2.2. Evaluation of Sequence Code Methods

Based on the constructed dataset of S, T, and Y phosphorylation sites, the prediction performance of different protein sequence coding methods under the self-attention capsule network is evaluated and compared by 5-fold cross-validation. The final comparison results are shown in Table 1.

For p-S and p-T sites, AAindex, AAglove, and GloVe achieve excellent performance, which verifies the ability of word embedding methods and physicochemical properties to capture information hidden in protein sequences. Different vector representations of protein fragments are generated by different word embedding techniques. GloVe has significant advantages over One-Hot encoding methods. The Acc, AUC, and MCC obtained by GloVe coding are 0.0215, 0.0175, and 0.0365 higher than those obtained by One-Hot coding, respectively. Compared to the sparse feature representations generated by One-Hot, GloVe can capture global statistical information and contextual information hidden in protein sequences through mapping phosphorylated and non-phosphorylated peptides truncated from high to low dimensional space. Moreover, physicochemical property coding shows advantages over word embedding techniques and hybrid feature coding in identifying S/T phosphorylation sites. The reason is that the physicochemical properties of amino acids are important factors influencing the formation of PTMs sites, which largely determine the diversity and specificity of protein structure and function [29].

For the p-Y sites, the average AUC and Acc achieved by the two physicochemical codes are 0.729 and 0.678, which are 0.003 and 0.017 higher than GloVe, respectively. The experimental results show that the accuracy of the prediction results obtained by word embedding is significantly lower than that of AAindex and AAPCA. Meanwhile, the results of AAglove are also lower than the test results of physicochemical coding. The main reason for this phenomenon may be that the physicochemical information and amino acid residue order of AAglove are compressed into the same dimension during the convolution process. Under the designed framework network structure, the best prediction performance is obtained for the physicochemical coding of amino acids in protein phosphorylation. The prediction results obtained by AAindex and AAPCA are similar, but the smaller amount of data introduced by AAPCA makes the prediction and training faster. Therefore, the AAindex coding method is used for the prediction problem of p-S and p-Y sites, and AAPCA coding is used for p-T sites.

### 2.3. Impact of Network Restructuring

Ablation experiments are performed to measure the improvement of the tuning on the capsule network. First of all, to measure the effectiveness of the routing mechanism tuning, we compared the model with CapsNET(Dynamic Routing). The only difference between EcapsP and CapsNET is the introduction of self-attention routing. The above two models are tested on a species-wide dataset of their own construction. The performance with the attention routing is superior to that with the dynamic routing, with the results of Acc, AUC, and MCC increasing from 0.598, 0.634, and 0.199 to 0.665, 0.727, and 0.352, respectively. To avoid the biased performance evaluation on a fixed test dataset, a 20% data set was randomly selected as an independent test set for testing, and the experiment is repeated 50 times. The average Acc for the 50 repeated experiments is improved by 7%. The introduction of self-attention routing has greatly improved the prediction accuracy of the model by getting closer to the true distribution of data while it has fewer parameters and is faster to train.

Second, unconditional reconstruction(No-Mask) improves the stability of the capsule network. In 50 repeated experiments at the p-Y site, the AUC of the unconditional reconstruction is higher than 0.72. The difference between the highest value of 0.74 and the lowest value of 0.69 for AUC in the Mask mechanism is 0.05. For the results on the independent test dataset, all the performance metrics of No-Mask are better than those of Mask except for Sp. The unconditional reconfiguration method replaces the Mask reconfiguration of the capsule network, and its contribution is to enhance the robustness of the system, resulting in a slight improvement in the performance of the novel capsule network.

Thirdly, we adjust the marginal loss (ML) function of the model to a cross-entropy loss (CEL) function, which is more suitable for binary classification problems. The adjustment of the loss function increases the Acc of EcapsP by 0.011. Besides, the average AUC and Sn of EcapsP (CEL) are 0.021 and 0.051, respectively, higher than EcapsP (ML). Especially, The MCC of EcapsP (CEL) is increased by 0.03 on average compared with EcapsP (ML). This suggests that the marginal loss function may not be an ideal objective function for phosphorylation site prediction. The addition of the residual structure (Short-Cut) also improves the identification ability of the model for this problem, especially for Acc, by 0.024 over the initial architecture. This indicates that the Short-Cut provided by the residual structure helps EcapsP to better fit the nonlinear relationship of the problem. All the results are shown in Table 2.

### 2.4. Comparison of Multiple Predictors

**Figure 2:**
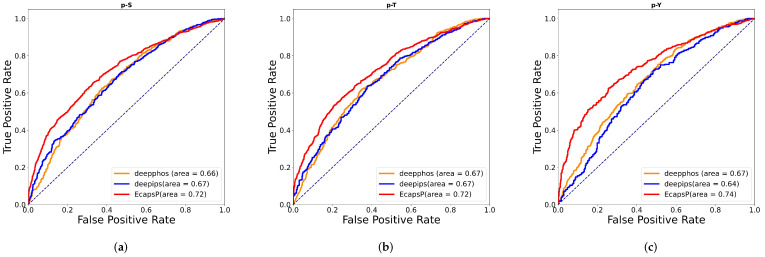
ROC curves of existing tools for phosphorylation site prediction on p-S, p-T, and p-Y independent test sets. (**a**) Serine phosphorylation, (**b**) Threonine phosphorylation, (**c**) Tyrosine phosphorylation. Notes: The ROC curve indicates the balance between sensitivity (or TPR) and specificity (1—FPR). Classifiers that bring the curve closer to the upper left corner indicate better performance.

To further evaluate the performance of EcapsP, we compared EcapsP with several available phosphorylation site predictors using the independent test dataset. Nevertheless, most models utilize different training data and do not provide separate tools or web servers, making it challenging to provide directed comparisons. End-to-end deep learning can automatically pick out salient features and discover complex representations of data patterns. Trained by the prokaryotic dataset, the deep learning-based predictors will be specific to prokaryotes. Therefore, to evaluate the prediction performance objectively, we compare our method with the previous three predictors based on deep learning (DeepIPS [30], CapsPTM [31], and DeepPhos [32]). Specifically, the comparison predictors are first rebuilt according to the original article. Then, we retrain these models on the same prokaryotic dataset as this study and test them on the same test set as this study. The results are presented in Table 3. For S, T, and Y sites, the Accs of EcapsP are 0.667, 0.664, and 0.688, which are 0.076, 0.07, and 0.078 higher than those of CapsPTM. Although CapsPTM is an outstanding model based on a capsule network, the comparison results show that EcapsP achieves a better performance overall. Compared with DeepPhos, our model improves Acc and AUC by 0.0497 and 0.0583, respectively. Meanwhile, the improvement of EcapsP on MCC is even more dramatic, with the MCC value upgraded from 0.253 to 0.333. Currently, DeepIPS is the most powerful predictor of general phosphorylation sites in human protein [30]. EcapsP outperforms DeepIPS in prediction performance on general phosphorylation sites in prokaryotes. For p-S, the Acc, AUC, and MCC of our model are improved by 0.046, 0.044, and 0.048, respectively, compared to DeepIPS. The prediction performance of EcapsP for p-T and p-Y also far exceeds that of DeepIPS. Figure 2 shows the ROC curves of the three better-performing methods. Obviously, the ROC curve of EcapsP is basically in the upper left of the other two methods, and the AUC is relatively large.

Subsequently, EcapsP is evaluated in comparison with other existing prokaryotic phosphorylation site prediction models. MPsite is the main comparison object [27], because it is the most advanced predictor and the only universal predictor that can predict phosphorylation in various prokaryotic. The specific model source code of MPsite is not yet open-source. To ensure fairness, EcapsP and several models developed based on deep learning are trained and tested by the dataset as MPsite. All results are shown in Table 4. For p-S site detection, the MCC value is 0.012 lower than MPsite. But, for Acc, Sn, and Sp, our model improved by 0.005, 0.045, and 0.01, respectively. It can be observed that when using the EcapsP to predict p-Y sites from MPsite, we obtained promising results with averages of Accuracy, AUC, and MCC as high as 0.765, 0.807, and 0.436. The new computational tool obtains better comprehensive performance with partially missing data in the training set, demonstrating that EcapsP is a more efficient predictor for general phosphorylation site identification in prokaryotes.

In conclusion, EcapsP provides a powerful predictor for prokaryotic phosphorylation sites. Meanwhile, the novel network model based on the capsule network structure provides a new alternative for the complex sequence classification problem.

### 2.5. Effectiveness Analysis of EcapsP for Small Datasets

To explore the strengths of our method (EcapsP) for small datasets, we compare it with CapsPTM, DeepIPS, DeepPhos, and CNN models on training sets with different sizes. The structure and complexity of the CNN model are similar to that of EcapsP, except that PrimaryCaps are converted to standard CNNs and DigiCaps are converted to fully connected layers. To avoid the issue of similar sequences affecting the evaluation of model performance, we use self-constructed non-redundant tyrosine phosphorylation samples as the benchmark dataset. The balanced dataset is constructed by combining positive phosphorylation samples (Y) and a randomly selected non-redundant subset of negative samples. In addition, we randomly select 20% of the balanced dataset as independent test data and use the remaining data as the original training dataset. 10%, 20%, 40%, 70%, and 100% of the samples in the original training dataset are randomly chosen to construct new training datasets, respectively.

**Figure 3:**
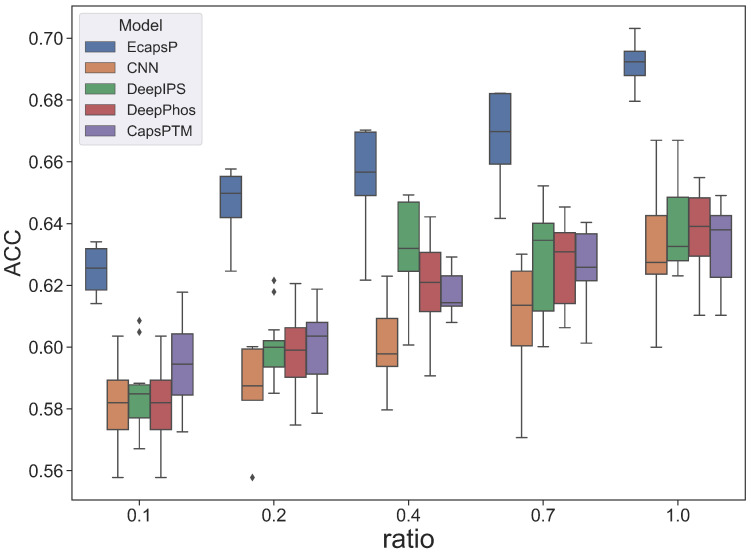
Under the training datasets with different sizes, boxplots of the ACCs achieved by EcapsP, CapsPTM, CNN, DeepIPS, and DeepPhos on the same independent test set. The X-axis represents the ratios of the number of samples in the new training datasets to that in the original training dataset. For each ratio, the simulation experiments are repeated 50 times.

On the same test data, the ACCs of prediction systems trained with the same new training datasets are plotted in Figure 3. To avoid bias from randomly selected samples, the simulation experiments for each ratio are repeated 50 times. For a fair comparison, amino acid sequences are transformed into numerical vectors by the same encoding method. As shown in Figure 3, EcapsP significantly outperforms other methods under the training datasets with different sizes, which demonstrates the capabilities of EcapsP to deal with the small dataset learning problem.

### 2.6. Performance of the EcapsP among Single Species and across Species

EcapsP has better cross-species validation performance than the previous method. However, the prediction accuracy under full species is still a huge deficiency compared to experimental biological tools. Considering that a variety of prokaryotes have evolved different protein kinases in diverse environments, training phosphorylation predictors for a single species has the potential to improve the accuracy of predictions. Experimental analyses are performed in a dataset of prokaryotic algae and several of their subspecies. From Table 5, the following points are observed.

After training on the ATCC33909 dataset, EcapsP predicts all three types of phosphorylation sites for this organism with over 80% accuracy. Compared to EcapsP trained under the full prokaryotic dataset, for p-S, p-Y, and p-T of ATCC33909, the MCC and AUC of EcapsP for single organisms are increased by 0.452 and 0.23 on average. In addition, the predictive performance of the model trained under the cyanobacterial phylum shows a decrease compared to training with ATCC33909 data alone. EcapsP trained under the all-prokaryotic algal category further reduces prediction accuracy. It can be tentatively concluded that the prediction accuracy increases in the case of biological phylum subdivision because the phosphorylation mechanism of organisms in the same phylum tends to be homogeneous. Moreover, after further subdivision, the prediction accuracy will continue to improve, indicating that the internal phosphorylation environments of different species in the same phylum also vary significantly.

The above results demonstrate that phosphorylation sites of prokaryotes should be predicted in the smallest possible phylum of organisms. However, most prokaryotes have only single-digit experimentally validated phosphorylation sites. It is necessary to use cross-species verification to explore the interrelationships between different species. EcapsP is trained with the p-S data of ATCC33909, and then the cross-species prediction performance of the model is tested with the p-S samples of ATCC35092 and ATCC27264, respectively. ATCC35092 and ATCC33909 are both cyanobacteria, while ATCC27264 is not a cyanobacterium but belongs to the same prokaryotic algae. On the test set of ATCC33909, and the p-S samples of ATCC35092 and ATCC27264, the Accs achieved by EcapsP are 0.816, 0.692, and 0.646, respectively. Experiments have demonstrated that the phosphorylation patterns of different organisms are dissimilar, and the difficulty of identifying biological phosphorylation sites across species is significantly increased.

### 2.7. Performance of the EcapsP on Eukaryote Phosphorylation

To verify the effectiveness of EcapsP on eukaryotes, we retrain and test EcapsP, DeepPhos [32], DeepIPS [30] and CapsPTM [31] on eukaryotic phosphorylation data. The training dataset and independent test datasets are constructed by human p-S p-T phosphorylation data and can be obtained from the open-source dataset of DeepIPS [30]. As listed in Table 6, EcapsP achieves the highest Acc and Sn. In terms of Sp, MCC, and AUC, EcapsP and DeepIPS achieve pretty close values and outperform the other methods. To sum up, EcapsP attains much more outstanding performance on eukaryote phosphorylation, which highlights its excellent generalization ability.

### 2.8. Visualization of Features

**Figure 4:**
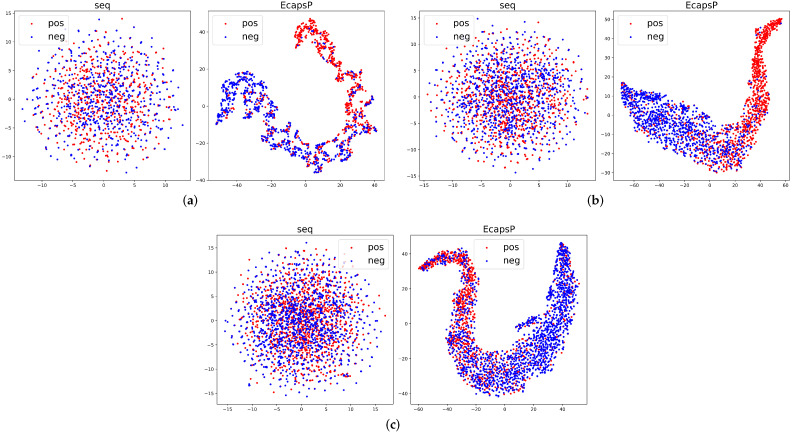
Visualization of the original sequence features and sequence features extracted by EcapsP. (**a**) Tyrosine phosphorylation. (**b**) Threonine phosphorylation. (**c**) Serine phosphorylation. The red point represents the phosphorylation samples while the blue point represents the unphosphorylation samples.

Deep learning techniques are widely criticized for their black box effect due to high nonlinearity. The interpretation of the principles is also a difficult problem for most deep learning-based models. We try to explain the deep mapping learned by EcapsP from a feature perspective. To demonstrate that EcapsP learns a more robust representation of phosphorylation, we use t-distributed stochastic neighbor embedding (t-SNE) [33] to visualize the original sequence features and the sequence features extracted by EcapsP for phosphorylation sites and non-phosphorylation sites. As plotted in Figure 4, the original features of phosphorylation and non-phosphorylation sites are mixed in disorder while the classification boundary of phosphorylation and non-phosphorylation sites in the feature space generated by EcapsP is clearly visible. These results suggest that the raw protein sequences can be transformed into meaningful representations by EcapsP.

## 3. Materials and Methods

### 3.1. Overview

**Figure 5:**
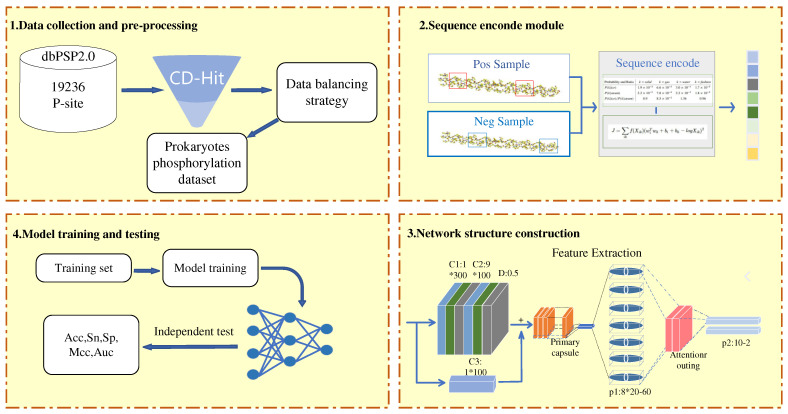
Flowchart of the EcapsP framework. The processes to develop EcapsP include 1. Creation of benchmark dataset, 2. Formulation of samples and calculations of feature vectors, 3. Construction and optimization of the prediction model, 4. Testing and validation of Model.

We construct a novel deep-learning framework aiming at predicting p-sites in prokaryotic proteins. The main process can be divided into four steps. In the first part, we build a benchmark dataset by collecting samples from dbPSP2.0. Protein sequence redundancy and data imbalance are solved by Cd-hit and EasyEnsemble algorithms. Subsequently, a sequence encoding module is constructed to convert the input amino acid peptide chain into a digital vector. Third, the appropriate classification algorithm is selected and the model details are changed according to the problem characteristics. Finally, the supervised model is trained on a baseline training set and evaluated on independent test data. The overall workflow is depicted in Figure 5.

### 3.2. Benchmark Dataset Construction

A phosphorylation benchmark dataset of prokaryotes is constructed by collecting annotations from dbPSP2.0. The database contains phosphorylation data for nearly 200 organisms with 19236 experimentally identified phosphorylation sites. As most features are difficult to obtain for many proteins except amino acid sequences, only protein sequence information is extracted to expand the application scope of the model [34]. The Cd-hit procedure is used to ensure that the sequence homology of the dataset is less than 70%, thereby reducing redundancy of phosphorylated protein sequences to avoid model overfitting [35]. The peptides with S/T/Y residues in the center and experimentally verified binding phosphate groups are positive samples. According to the convention in most studies, a negative sample needs to meet two criteria. First, the site cannot be found as a positive sample. Second, it must occur on a protein that has at least one confirmed positive site. Each sample is denoted as a peptide segment of 33 (±16) residues centered on S/T/Y. This method obtains a large number of negative samples that do not match the number of positive samples, and the specific information is shown in Table 7. To counteract the negative effects of unbalanced data sets, the Easy Ensemble strategy is introduced to balance positive and negative sample data. To make fair companions with existing methods, MPsite’s training set and independent test set are downloaded to validate the model performance. In addition, a dataset of prokaryotic algal organisms is constructed to verify the differences in phosphorylation patterns between species.

### 3.3. Sequence Encoding Strategies

**One-Hot Encoding**

Before the advent of one-hot coding, machine learning classification algorithms could not directly deal with disordered and discrete features. The reason for this phenomenon is that classifiers usually deal with continuous and ordered data. Using One-Hot Encoding, the values of discrete features are extended to Euclidean space. A value of a discrete feature corresponds to a point in Euclidean space. This approach uses a 21-bit status register to encode 20 amino acid residues and a null site, with each state corresponding to its own register bit. At any time just one of the 21 registers is valid, meaning that only one bit is a 1 and the rest are 0. However, when many feature classes exist, the data may become too sparse after one-hot encoding.

**GloVe**

Word embedding technology is a general term for language modeling and learning technical expressions in natural language processing (NLP). It embeds a high-latitude space with all word count dimensions into a continuous vector space with low-latitude dimensions. The studies have shown that using a word embedding to represent fixed-length DNA sequences yields satisfactory prediction performance in the fields of promoters and enhancers [36]. GloVe is an unsupervised learning algorithm for generating word vector representations [37]. It uses the co-occurrence matrix to consider both local information and overall information. This approach combines the advantages of two main approaches in word embedding processing: the use of global statistical information from the corpus and local contextual features.

**6D Physical-Chemical Codes**

A 6-dimensional vector is applied to represent each amino acid, where the first 5 dimensions are the combination of physicochemical properties obtained by principal component analysis(PCA) and the other 1D represents the absence of amino acid gaps in the 33 length segments [31]. The representation of physicochemical properties is derived from the quantitative representation of amino acids proposed by Venkatarajan et al. [38]. This approach uses five main components to reproduce the main variations of 237 properties of 20 amino acids. When the length of the peptide fragment is less than 33, the five principal components of the redundant positions are set to 0 and the remaining 1 position is set to 1. On the other hand, the last 1D is set to 0. This approach reduces the data dimension to accelerate the calculation. AAPCA is used later to represent this approach.

**10D Physical-Chemical Codes**

Protein sequences consist of 20 amino acids with different physicochemical properties. The physicochemical properties of amino acids have long been pivotal in protein-related research [39]. The Amino Acid Index is a database containing numerical quantification of the different physicochemical properties of amino acids [40]. In this study, 20 amino acid residues are quantified according to the normalized results of nine properties of AAIndex, including amino acid composition, average flexibility index, hydrophobicity index, net charge, partition coefficient, residue volume, molecular weight, the accessible surface area of residues, and accessible surface area. The tenth dimension indicates whether there is a gap in the current amino acid position.

**AAglove**

AAglove introduces both protein physicochemical information and residue information. This approach uses a two-dimensional array to represent amino acid residues. The representational space of each residue extends two dimensions, one of which is its physicochemical information encoding and the other is the corresponding GloVe encoding. Physicochemical information is used to construct 10D physical-chemical codes. The GloVe in the other dimension is also a 10-dimensional vector, ensuring that the data from the two channels can be spliced or accumulated by convolution of the same size.

### 3.4. The Framework of the Proposed EcapsP

The model consists of two main modules: (A) Sequence encoding module; (B) Network module. The overall process is as follows.

**Sequence Encoding Module**

Firstly, the sequence encoding module extracts fragments of biological protein sequences with potential phosphorylation sites. Next, the intercepted protein fragments are segmented into protein subword sequences using an n-gram approach. Subsequently, a fixed vector representation of the protein subword is generated according to the corresponding sequence encoding method. Finally, the vector representing the protein sequence is transferred to a network module.

**Network Module**

**Figure 6:**
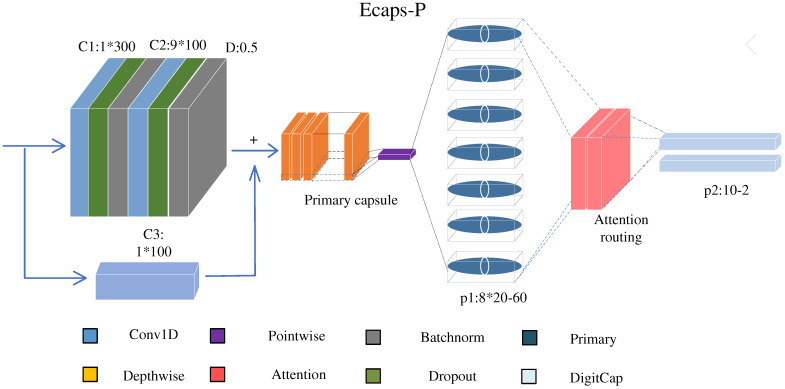
The novel capsnet architecture consists of CNN and self-attention routing. The CNN is used to extract high-level motif features containing interconnections and mutual position relationships among features. The BatchNorm layer and dropout layers are interspersed in the CNN to solve the overfitting problem. The self-attention layer is used to obtain remote dependencies between residues in protein sequences [41].

Figure 6 illustrates the pipeline of the proposed EcapsP.

**(a) Convolutional layer:**

This layer converts protein-encoding information into local feature detector activity, where each filter is convolved with the input of the layer to encode local knowledge of small sensory fields. Specifically, the first Conv layer has 300 size-1 1D convolution kernels with a stride of 1 and the second layer has 100 size-9 1D convolution kernels with a stride of 1, both using ReLU as the activation function [42]. In summary, the first part of the network is modeled as a single function, Conv, which maps the input sequence information to a higher-dimensional space, thus facilitating the creation of capsules.

**(b) Dropout + Batchnorm:**

The purpose of this part is to reduce overfitting by introducing noise into the network. Dropout is a primary technique for regularization. The probability of dropout is adjusted to 50%. Batch normalization regresses the input distribution of the neural network to the standard distribution. Its main role is to avoid the vanishing gradient problem. Moreover, since Batchnorm has specialized scaling and offset factors, it can effectively match the filtering effect of the relu function [43].

**(c) Convolutional residual block:**

Phosphorylation site determination for multiple organisms is a complex problem. Simple CNN models are difficult to fit such complex nonlinear relationships, and deepening the network may lead to the problem of gradient disappearance [44]. According to the residual structure in the idea of ResNet, a shortcut is added to the model, which can dynamically adjust the complexity of the model.

**(d) Primary capsules:**

Each capsule uses a convolution filter to convert the local knowledge of the small receptive field into a vector representation of the features. In our model, PrimaryCaps is a new type of convolutional capsule layer. Unlike the original convolutional capsule layer, PrimaryCaps consists of a deeply divisible convolutional block. The first part of the divisible convolution block is a channel-by-channel convolution, and the second part is a point-by-point convolution. This section converts the output of the convolution layer into 60 primary capsules, each containing 8 convolution units. The length of a capsule in a capsule network represents the probability of the appearance of its corresponding entity [45].

**(e) Self-attention routing:**

The digital capsules generated by dynamic routing lack information about the remote dependencies between protein sequence residues, and dynamic routing mechanisms are likely to reduce the robustness of the system [46]. The batch dot product method is used to transform the dynamic routing into self-attention routing. In this way, EcapsP captures not only the local patterns of protein sequences but also the long-term dependence of protein sequences, enhancing the robustness of the system. The specific computations are explained in Algorithm 1.

The routing layer encodes the primary capsules as two 10-unit numeric capsules. The lengths of the digital capsules in the capsule network represent the probability of the occurrence of their corresponding entities [45]. Unlike the traditional softmax classification, the capsule network predicts the length of the two vectors as the probability of phosphorylated and unphosphorylated sites, respectively. EcapsP uses a new activation function that compresses the length of the capsule between [0, 1] [47].


**Algorithm 1** Attention Routing using Scalar Product **Input:** input parameters capsule $u_{n}^{l}$ from layer *l* **Output:** output the Digicaps: $V_{j}$
  1: affine transformation for all $u_{n}^{l}$:

$${\hat{U}}_{\left(n^{l},n^{l+1},d^{l+1}\right)}^{l}=u_{n}^{Tl}\times W_{\left(n^{l},n^{l+1},d^{l},d^{l+1}\right)}^{l}$$


  2: Calculating self-attention weights:

$$A_{\left(n^{l},n^{l},n^{l+1}\right)}^{l}=\frac{{\hat{U}}_{\left(n^{l},n^{l+1},d^{l+1}\right)}^{l}\times {\hat{U}}_{\left(n^{l},n^{l+1},d^{l+1}\right)}^{Tl}}{\sqrt{d^{l}}}$$


  3: Use softmax to Calculate Weights *C*:

$$C_{\left(n^{l},n^{l+1}\right)}^{l}=\frac{exp(\sum_{n^{l}}A_{\left(n^{l},n^{l},n^{l+1}\right)}^{l})}{\sum_{n^{l+1}}exp(\sum_{n^{l}}A_{\left(n^{l},n^{l},n^{l+1}\right)}^{l})}$$


  4: For all capsule *j* in l+1:

$$s_{n}^{l+1}={\hat{U}}_{\left(n^{l},n^{l+1},d^{l+1}\right)}^{l}\times \left(C_{\left(n^{l},n^{l+1}\right)}^{l}+B_{\left(n^{l},n^{l+1}\right)}^{l}\right)$$


  5: Compress the capsule length to between 0 and 1:

$$V_{j}\left(x_{1}\ldots x_{m}\right)\leftarrow squash\left(s_{n}^{l+1}\right)$$


  6: **return**
$V_{j}$



Algorithm 1 Explain how the data stream flows in digicaps. $W_{\left(n^{l},n^{l+1},d^{l},d^{l+1}\right)}^{l}$ is the affine matrix used to map the main capsule layer input to a higher dimensional space. $B_{\left(n^{l},n^{l+1}\right)}^{l}$ is the introduced deviation. $V_{j}$ is the final output generated by the self-attention routing.

**(f) Decoding layer:**

**Figure 7:**
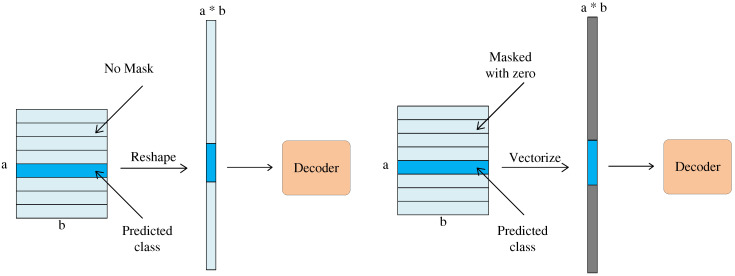
Mechanisms for conditional and unconditional decoders. The blue rectangular block represents the longest vector, and the gray color indicates that the vector is replaced by an all-zero vector.

Reconfiguration regularization is a simple three-layer fully connected network. The Capsule network uses a masking mechanism in the decoding step, which masks all activities except the correct activity vector. This activity vector is then used to reconstruct the sequence encoding. However, unconditional reconstruction rather than a masking mechanism is used in this work to encourage capsules to encode the instantiation parameters of the input sequence. Unconditional reconstruction uses all vectors of Digicaps to reconstruct the input. The details of the two reconfigurations are shown in Figure 7. The goal is to minimize the difference between the decoder output and the protein sequence encoding [48].

### 3.5. Performance Assessment

Five performance measures, accuracy (Acc), sensitivity (Sn), specificity (Sp), Matthew correlation coefficient (MCC), and area under the ROC curve (AUC), are used to assess the discriminatory ability of the proposed method. The first four measures are defined as

(1) $$Acc=\frac{TP+TN}{TP+TN+FN+FP}$$

(2) $$Sn=\frac{TP}{TP+FN}$$

(3) $$Sp=\frac{TN}{TN+FP}$$

(4) $$MCC=\frac{TP\times TN-FP\times FN}{\sqrt{\left(TN+FN)\times (TP+FP)\times (TN+FP)\times (TP+FN\right)}}$$

where TP, TN, FP, and FN represent true positives, true negatives, false positives, and false negatives, respectively.

## 4. Conclusions and Discussion

Phosphorylation of prokaryotes is closely associated with the generation of various superbugs, and its study plays an imperative role in advancing the development of novel antibacterial drugs. Limited by time and funding for biological experiments, a suitable and effective computational method is urgent to be developed to identify phosphorylation in prokaryotes. In this study, EcapsP is proposed to predict phosphorylation sites. Firstly, the non-redundant prokaryotic phosphorylation datasets are built based on Cd-Hit technology. Then, the capsule network is improved using self-attentive mechanisms and shortcuts that enable it can capture both short-range and long-range relevant information. Finally, the novel capsule network was used to construct a phosphorylation site prediction model. The results show that EcapsP has better capabilities than existing prokaryotic phosphorylation predictors. In addition, the model is the first predictor to provide detection of prokaryotes tyrosine phosphorylation. The obvious drawback of neural network models is the lack of interpretability. In future work, we will use other explanatory neural networks, such as tabular learning networks (TabNet) and additive exponential models (xNN), to explore the phosphorylation patterns. Furthermore, the currently used EasyEnsemble has a limited effect in handling imbalances in phosphorylated datasets. In future research, it may be possible to explore the feasibility of GAN networks in solving such problems.

## Figures and Tables

**Table 1:** Prediction performance of different protein sequence coding methods by the 5-fold cross-validation.

Residue Type	Method	Acc	Sn	Sp	MCC	AUC
S	One-Hot	0.634	0.697	0.574	0.273	0.686
	GloVe	0.641	0.661	**0.617**	0.278	0.7
	AAindex	**0.667**	**0.727**	0.608	**0.338**	**0.721**
	AAPCA	0.649	0.691	0.595	0.289	0.701
	AAglove	0.615	0.709	0.517	0.232	0.664
T	One-Hot	0.62	**0.771**	0.469	0.251	0.677
	GloVe	0.656	0.766	0.545	0.319	0.698
	AAindex	0.653	0.662	**0.635**	0.302	0.702
	AAPCA	**0.661**	0.742	0.584	**0.33**	**0.722**
	AAglove	0.635	0.759	0.508	0.276	0.682
Y	One-Hot	0.626	0.709	0.556	0.259	0.679
	GloVe	0.661	0.723	0.609	0.324	0.726
	AAindex	**0.681**	0.736	**0.624**	0.366	**0.734**
	AAPCA	0.674	**0.871**	0.472	**0.374**	0.725
	AAglove	0.632	0.66	0.603	0.264	0.676

**Table 2:** Prediction results of ablation experiments.

Type	Method	Acc	Sn	Sp	MCC	AUC
Decode	Mask	0.677	0.735	**0.613**	0.332	0.723
	No Mask	**0.679**	**0.756**	0.604	**0.349**	**0.73**
Loss Function	ML	0.656	0.684	**0.628**	0.312	0.712
	CEL	**0.667**	**0.735**	0.613	**0.342**	**0.733**
Network	No Short-Cut	0.652	0.724	0.582	0.307	0.698
	Short-Cut	**0.676**	**0.735**	**0.622**	**0.35**	**0.729**
Routing	Dynamic Routing	0.598	0.34	0.455	0.199	0.634
	Self-Attention	**0.665**	**0.727**	**0.604**	**0.352**	**0.727**

**Table 3:** Prediction performance of various deep learning models on a benchmark dataset of prokaryotic phosphorylation sites.

Residue Type	Method	Acc	Sn	Sp	MCC	AUC
S	CapPTM [31]	0.591	0.528	0.604	0.182	0.631
	DeeIPS [30]	0.621	0.563	**0.649**	0.254	0.673
	DeepPhos [32]	0.625	0.578	0.571	0.25	0.664
	EcapsP	**0.667**	**0.735**	0.613	**0.302**	**0.717**
T	CapPTM [31]	0.594	0.418	0.706	0.162	0.626
	DeeIPS [30]	0.627	0.458	**0.733**	0.255	0.671
	DeepPhos [32]	0.629	0.619	0.637	0.257	0.668
	EcapsP	**0.664**	**0.694**	0.633	**0.328**	**0.721**
Y	CapPTM [31]	0.61	0.17	0.619	0.235	0.621
	DeeIPS [30]	0.605	0.435	**0.761**	0.25	0.636
	DeepPhos [32]	0.616	0.371	0.402	0.252	0.669
	EcapsP	**0.688**	**0.717**	0.645	**0.367**	**0.738**

**Table 4:** Prediction performance of various models based on MPsite dataset. Due to the lack of open source models, the experimental data for MPsite and NetphosBac in the table are taken from the Md’s paper [27].

Residue Type	Method	Acc	Sn	Sp	MCC	AUC
S	CapPTM [31]	0.58	0.372	0.669	0.04	0.609
	DeepIPS [30]	0.656	**0.818**	0.346	0.173	0.642
	NetphosBac [25]	0.562	0.331	0.678	−0.006	*
	Mpsite [27]	0.678	0.412	0.811	**0.239**	*
	EcapsP	**0.683**	0.457	**0.821**	0.227	**0.695**
T	CapPTM [31]	0.622	0.427	0.328	0.167	0.616
	DeepIPS [30]	0.707	**0.769**	0.567	0.33	0.745
	NetphosBac [25]	0.562	0.331	0.678	−0.006	*
	Mpsite [27]	0.751	0.616	0.818	0.432	*
	EcapsP	**0.765**	0.567	**0.855**	**0.436**	**0.807**

The * in the table means that data is not available here.

**Table 5:** Prediction results under different biological phyla.

Residue Type	Class	Acc	Sn	Sp	MCC	AUC
S	prokaryote	0.667	0.735	0.613	0.302	0.713
	nuclear algae	0.702	0.738	0.664	0.403	0.758
	cyanophyte	0.757	0.866	0.655	0.514	0.797
	ATCC 33909	0.845	0.875	0.753	0.548	0.872
T	prokaryote	0.659	0.695	0.613	0.31	0.7
	nuclear algae	0.721	0.665	0.772	0.44	0.763
	cyanophyte	0.777	0.886	0.785	0.537	0.813
	ATCC 33909	0.856	0.905	0.80	0.712	0.912
Y	prokaryote	0.688	0.815	0.558	0.386	0.738
	nuclear algae	0.718	0.758	0.682	0.439	0.801
	cyanophyte	0.736	0.775	0.698	0.459	0.805
	ATCC 33909	0.807	0.865	0.695	0.568	0.853

**Table 6:** Prediction performance of various deep learning models on a test dataset of eukaryotic phosphorylation sites.

Method	Acc	Sn	Sp	MCC	AUC
CapPTM [31]	0.802	0.789	0.815	0.604	0.876
DeepIPS [30]	0.806	0.796	**0.835**	**0.632**	**0.883**
DeepPhos [32]	0.792	0.773	0.831	0.602	0.883
EcapsP	**0.812**	**0.809**	0.828	0.629	0.889

**Table 7:** The specific information of the benchmark dataset generated by Cd-hit for dbPSP2.0.

	S	T	Y
Pos	6629	5029	3167
Neg	168,644	122,985	47,375
Substrate	4231	3297	2267

## Data Availability

Not applicable.
